# Real-life and real-time hearing aid experiences: Insights from self-initiated ecological momentary assessments and natural language analysis

**DOI:** 10.3389/fdgth.2023.1104308

**Published:** 2023-03-15

**Authors:** Charlotte Vercammen, Ilze Oosthuizen, Vinaya Manchaiah, Pierre Ratinaud, Stefan Launer, De Wet Swanepoel

**Affiliations:** ^1^ Sonova AG, Research & Development, Stäfa, Switzerland; ^2^ Manchester Centre for Audiology and Deafness, School of Health Sciences, Faculty of Biology, Medicine and Health, University of Manchester, Manchester, United Kingdom; ^3^ Department of Speech-Language Pathology and Audiology, University of Pretoria, Pretoria, South Africa; ^4^ Virtual Hearing Lab, Collaborative initiative between University of Colorado, School of Medicine, Aurora, CO, USA, and University of Pretoria, Pretoria, South Africa; ^5^ Department of Otolaryngology–Head and Neck Surgery, University of Colorado School of Medicine, Aurora, CO, United States; ^6^ UCHealth Hearing and Balance, University of Colorado Hospital, Aurora, CO, United States; ^7^ School of Allied Health Sciences, Department of Speech and Hearing, Manipal Academy of Higher Education, Manipal, India; ^8^ Laboratoire D'Études et de Recherches Appliquées en Sciences Sociales (LERASS), University of Toulouse, Toulouse, France; ^9^ Sonova AG, Audiology & Health Innovation, Stäfa, Switzerland; ^10^ School of Health and Rehabilitation Sciences, The University of Queensland, Brisbane, QLD, Australia; ^11^ Ear Science Institute Australia, Perth, WA, Australia

**Keywords:** hearing-aids, ecological momentary assessment (EMA), natural language analysis, real-world data, mobile applications (apps), data logging, personalized care, computational audiology

## Abstract

**Introduction:**

Smartphone technology can provide an effective means to bring real-life and (near-)real-time feedback from hearing aid wearers into the clinic. Ecological Momentary Assessment (EMA) encourages listeners to report on their experiences during or shortly after they take place in order to minimize recall bias, e.g., guided by surveys in a mobile application. Allowing listeners to describe experiences in their own words, further, ensures that answers are independent of predefined jargon or of how survey questions are formulated. Through these means, one can obtain ecologically valid sets of data, for instance during a hearing aid trial, which can support clinicians to assess the needs of their clients, provide directions for fine-tuning, and counselling. At a larger scale, such datasets would facilitate training of machine learning algorithms that could help hearing technology to anticipate user needs.

**Methods:**

In this retrospective, exploratory analysis of a clinical data set, we performed a cluster analysis on 8,793 open-text statements, which were collected through self-initiated EMAs, provided by 2,301 hearing aid wearers as part of their hearing care. Our aim was to explore how listeners describe their daily life experiences with hearing technology in (near-)real-time, in their own words, by identifying emerging themes in the reports. We also explored whether identified themes correlated with the nature of the experiences, i.e., self-reported satisfaction ratings indicating a positive or negative experience.

**Results:**

Results showed that close to 60% of listeners' reports related to speech intelligibility in challenging situations and sound quality dimensions, and tended to be valued as positive experiences. In comparison, close to 40% of reports related to hearing aid management, and tended to be valued as negative experiences.

**Discussion:**

This first report of open-text statements, collected through self-initiated EMAs as part of clinical practice, shows that, while EMA can come with a participant burden, at least a subsample of motivated hearing aid wearers could use these novel tools to provide feedback to inform more responsive, personalized, and family-centered hearing care.

## 1. Introduction

Hearing aids are the first line intervention method for managing sensorineural hearing loss (1) and have been shown to improve audibility (2), communication (1), and hearing-related quality of life (3, 4).

After an initial hearing aid fitting, during which amplification parameters are set to prescription targets according to a fitting formula – very often relying on the audiogram as input (5) – hearing aids can be further fine-tuned to a listener's preferences. Clinicians often depend on their expert knowledge to do so (6), especially in response to experiences reported by listeners during a follow-up appointment (7). By then, listeners have typically been wearing the hearing aids for a couple of days or weeks following the initial fitting. During this time, listeners experience aided hearing in their own natural environments and become more accustomed to using the new technology. Typically, listeners would identify situation-specific hearing events during such trial periods, especially challenging situations, remember them, and report them to the clinician during the next appointment. Clinicians, in turn, rely on the listeners' descriptions to interpret these hearing events (6, 7), identify in which acoustic environments they might have occurred, and what fine-tuning actions could potentially resolve the challenges experienced (5).

It is unsurprising that clinicians and hearing aid wearers might use different terminology to describe similar hearing events. To overcome such communication barriers, previous research has aimed to define a common lexicon that could be used during hearing rehabilitation by all parties involved. Gabrielsson and Sjögren (1979) (8) asked listeners with normal hearing to provide “free verbal descriptions” when listening to sound samples recorded through hearing aids, in order to construct a catalogue of perceptual sound quality dimensions. Other research has relied on clinician reports of how listeners with hearing loss describe hearing aid fitting-related problems and what actions they would perform to resolve them (7, 9). Still, the process of fine-tuning remains complex and very often iterative (5). Even when using pre-defined sound quality descriptors in a laboratory setting, there seems little consensus among listeners with hearing loss when asked to assign them to their listening experiences (10). Also, listeners typically report on their experiences a couple of days or weeks after the event, i.e., during their next follow-up appointment with a clinician. Such delayed reports might be particularly prone to recall (memory) biases when describing fluctuating perceptual experiences (11, 12), such as those experienced during a hearing aid trial. Further, hearing aid wearers might report on experiences other than sound quality-related matters during a follow-up appointment. Concerns related to handling, maintenance, repairs, information and training needs, physical fit and comfort, experienced benefit, and personal experiences or attitudes have also been described (13, 14).

Recently, smartphone-connected hearing aids have allowed listeners to report on their experiences in (near-)real time, i.e., during or shortly after they occur, by responding to surveys in a mobile application. This type of repeated data collection, also referred to as “experience sampling” or Ecological Momentary Assessment (EMA (15, 16)) aims to reduce recall bias by condensing the time between the experience and the report. As EMA has become increasingly common in hearing research (for an overview, see (17)), it has also been proposed as a useful tool in clinical practice (17, 18). While EMAs can be sampled at certain times, time intervals, or upon detection of specific parameters (e.g., through the hearing aid microphone), EMAs can also be self-initiated. In the latter case, participants decide when an event of interest takes place and report on their experiences by manually accessing a survey (17). Allowing listeners to describe experiences in their own words, e.g., through an open-text field opposed to selecting an answer from multiple choice options, can ensure that answers are independent of predefined jargon or of how survey questions are formulated. Accordingly, listeners can provide detailed information to their clinician, who, in turn, can access these reports, e.g., in the fitting software, and interpret them together with technical or acoustical parameters read out from the hearing aid, thereby fostering more responsive and personalized hearing care. Large-scale, real-world data sets collected in this manner would also facilitate training of machine learning algorithms, which could foster hearing technology to anticipate user needs.

Here, we describe a retrospective, exploratory analysis of clinical data, collected through EMA. The dataset consists of self-reported satisfaction ratings (indicating whether a momentary experience with hearing technology was positive or negative) and written open-text statements describing the experiences. Both were provided by real-world hearing aid wearers as part of their hearing care, by self-initiating surveys on a mobile application. Our aim was to explore how listeners describe their experiences with hearing technology, in real-time and in their own words, and identify emerging themes in the reports. Also, we explored whether identified themes correlated with the nature of the experiences, i.e., positive vs. negative self-reported satisfaction ratings. We hypothesized hearing aid wearers to describe perceptual experiences and experiences with hearing technology, with guidance for further fine-tuning, as well as related to practical matters, e.g., in terms of handling of the hearing devices (13, 14). As the EMAs were self-initiated, we hypothesized listeners to rely on the tool mostly to request help from a clinician, report problems, or negative experiences. To the best of our knowledge, this is the first report of a large, real-world EMA dataset that was collected clinically, as part of adult hearing care. Also, to the best of our knowledge, it is the first report of a substantial amount of open-field, written text samples that were collected through self-initiated EMAs.

## 2. Materials and methods

### 2.1. Study design

A total sample of 8,793 self-initiated EMAs, provided by 2,301 hearing aid wearers as part of their hearing care, were analyzed retrospectively. The de-identified EMA data was collected through a smartphone mobile application compatible with commercially available hearing aids. No demographic data was collected, other than country in which the hearing aids were adjusted, as well as brand and type of the hearing aids – from which the technology level of the devices could be deducted. All listeners were lawfully informed that their de-identified data could be analyzed for clinical and research purposes upon acknowledging the data privacy notice of the mobile application. Ethical approval was obtained from the Institutional Review Board at Lamar University, Beaumont, Texas, United States (IRB-FY21-252) prior to analyzing the data.

### 2.2. Data collection

The smartphone mobile application through which the data was collected, was freely available for download in the app store. The particular feature of the app through which the data was collected, was accessible for hearing aid wearers when it was activated by a clinician in the fitting software. Clinicians could choose to activate the feature whenever they believed that the listener and their hearing rehabilitation would benefit from a real-time feedback system, e.g., to highlight situations relevant for further counselling or fine-tuning. Listeners were required to open the mobile application on their own initiative, when they decided that an event of interest took place (self-initiated EMA), and (1) indicate whether they were having a positive or negative experience (multiple choice, further referred to as self-reported satisfaction rating); (2) respond to one, sometimes two (depending on the answers provided by the listener) multiple choice questions related to the momentary listening environment and problems encountered in these environments (not considered for this report); (3) optionally, add additional feedback through an open-text field, by describing their experiences in their own words.

### 2.3. Data extraction and data preprocessing

**Figure 1:**
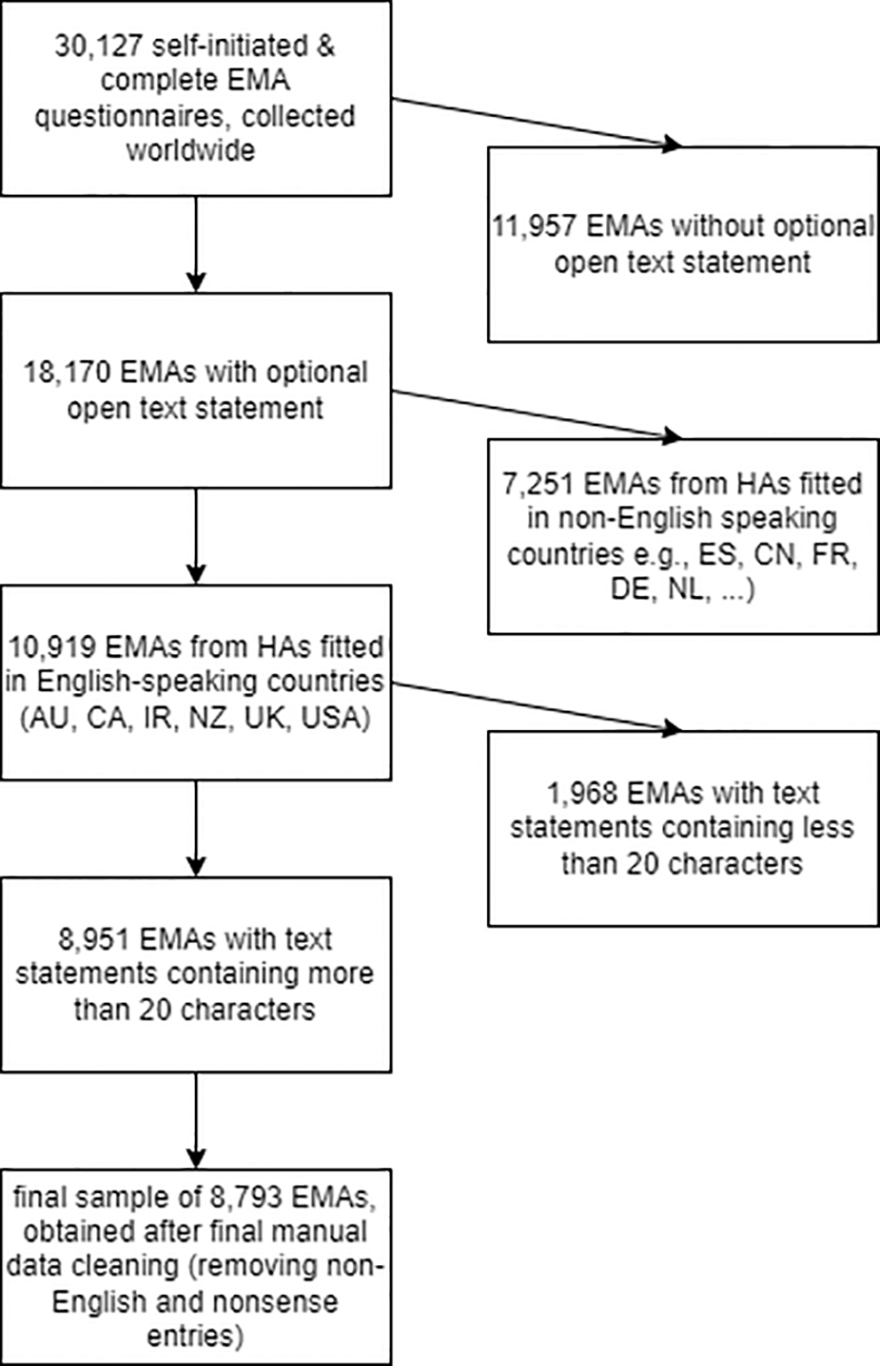
Flow chart visualizing how the final sample of 8,793 EMAs considered for further analysis was obtained.

An initial sample of 30,127 self-initiated EMAs on real-world hearing aid experiences was collected worldwide between May 2018 and June 2021 and extracted from cloud-based data logging of the smartphone mobile application. The data was preprocessed using R statistical software (19). As visualized in Figure 1, only EMAs containing an optional open text statement (18,170 EMAs, or 60% of the original sample) and EMAs collected from hearing aids fitted in English-speaking countries (i.e., Australia, Canada, England, Ireland, New Zealand, United States) were considered for further analysis. Text statements shorter than 20 characters were removed to eliminate entries with little content, after which a further manual data cleaning ensured that spelling mistakes were corrected, nonsense text, and a couple more non-English entries were removed. This last process resulted in a final dataset of 8,793 EMAs considered for further analysis.

### 2.4. Data analysis

The final sample of 8,793 self-initiated EMAs was analyzed using R statistical software (19) and Iramuteq [version 0.7, alpha 2] (20), an interface of R statistical software (19) for automated text analysis.

To explore how listeners describe their experiences with hearing technology, a cluster analysis was performed on the open text data, in order to identify emerging themes in the reports. The cluster analysis method applied here was a Descending Hierarchical Classification using the Reinert Method (21, 22). This method was chosen as it is an automated text analysis method that allows to process large amounts of text data in a time-efficient way, while at the same time aiming for similar goals as classical (manual) qualitative text analysis methods (23). The method considers segments of text as textual units and is based on the principle that words that occur together are related to each other, in terms of semantic meaning (reflecting topics or themes) or context (reflective of a certain period in time, geographic location, or socio-demographic traits of the speaker or writer) (24). Thereby, the Reinert Method (21, 22) not only allows identification of themes in a text corpus, but also reveals what themes are more or less related. The method has been used in a variety of research fields, such as sociolinguistics (24, 25), political sciences (26, 27), medicine (28, 29), and recently, also in audiology (30–34).

While the Reinert Method (21, 22) is a predominantly unsupervised classification algorithm (24), it requires some input from the researcher, such as the final number of clusters to be obtained (also see next paragraph). By default, the algorithm segments the text corpus considered into smaller textual units, which typically contain 35 to 40 words. The algorithm takes into account punctuation to obtain the text segments. When one considers a book as a text corpus, the textual units would approximately correspond to sentences. For a corpus consisting of naturally short texts statements such as considered here, i.e., one text corresponding to one written feedback from one listener, further segmentation is not required. The algorithm, then, lemmatizes all word forms considered, i.e., the word forms are transformed to their most basic, dictionary form. Afterwards, the text corpus is converted to a matrix. The matrix consists of one row for every textual unit that makes up the total corpus and one column for every adjective, noun, adverb, and verb that is present in the corpus. Each cell contains a value “1” (when the textual unit indicated by the row contains the word form indicated by the column) or “0” (when the textual unit indicated by the row does not contain the word form indicated by the column). For a simplified example, see Ratinaud (22). The algorithm then splits the matrix into smaller matrices, each split relying on a factorial correspondence analysis (22), a statistical method that summarizes a dataset along two dimensions based on the Pearson Chi-square (*χ*^2^) statistic. Hereby, the *χ*^2^-value reflects the association between the rows and columns of a contingency table and *χ*^2^/*n* (with n the grand total of the contingency table) the association strength between two categorical variables (for a review on factorial correspondence analysis, see (35) – note that *χ*^2^/*n* applies only to the simple use case of a two-way contingency table). For the first split, the algorithm divides the initial matrix into two sub-matrices, which are as lexically different from each other as possible. This is done by minimizing the number of word forms that the two sub-matrices have in common (23), or in terms of the factorial correspondence analysis underlying it, by maximizing *χ*^2^/*n* for the contingency table calculated by summing the two resulting sub-matrices (22). The split is then iteratively optimized by moving each row from the main matrix to the other sub-matrix than the one it ended up in and recalculating *χ*^2^/*n*. The change is kept, whenever it increases *χ*^2^/*n*, and reverted when it decreases *χ*^2^/*n*. This process is repeated until no more changes increase *χ*^2^/*n*. In this first step, the algorithm has identified the two most distinctive clusters in the corpus, i.e., two lexical themes that are as different from each other as possible (23). Afterwards, the algorithm relies on recursive bipartitions, i.e., each of the two lexical themes is again split into two new clusters based on a similar approach as outlined above, after which the largest of the remaining clusters is split into two clusters. This last process of further splitting the largest remaining cluster is repeated until a predefined number of final clusters is obtained (22). The result of the descending hierarchical classification is a number of lexical themes or clusters, each characterized by a list of words that have the strongest association, i.e., the highest *χ*^2^ values, with each respective cluster. For an example of how *χ*^2^ is calculated for individual word forms, see Pélissier (36, p. 35). The Descending Hierarchical Classification will further be referred to as cluster analysis.

Concretely, the data set considered here was first pre-processed to match the required data format for Iramuteq [version 0.7, alpha 2] (20), as per the available instructions (36, 37). In the second step, textual units were defined. For the current dataset, one text statement, reflecting one written feedback from one hearing aid wearer, was considered as one textual unit. As the statements were short in nature, the texts were not further segmented into smaller units. To avoid further segmenting while running the analysis, the “make text segments” option can be deactivated when importing the text corpus. Further, the “simple on texts” option can be chosen when running the clustering in Iramuteq (20). A challenge with any automated clustering is deciding how many topics will be considered (23). For this analysis, the “number of terminal clusters on phase 1” was increased from 10 (default setting) to 20. It is important to stress the exploratory nature of the analysis. Clustering is an iterative approach: when there is too much overlap between two resulting clusters and they cannot be distinguished from each other by the researcher or domain expert, the number of clusters can be decreased. When a certain cluster captures too much content according to the researcher or domain expert, the number of clusters can be increased to explore whether multiple themes were captured by the same cluster. Also, it is important to note that the algorithm creates the number of clusters requested, but only keeps clusters of a certain size. Under the default settings, the minimum size required to keep a cluster is determined by the number of text (segments) in the total text corpus, divided by the number of clusters requested. Also, the “maximum number of analyzed forms” was increased from 3,000 (default setting) to 30,000. As we had a reasonably large data set, it ensures that the algorithm considered all individual word forms (other than those with a frequency < 3) available in the text corpus. Based on the output of the algorithm, it is the researcher's task to interpret the clusters and name them, based on a list of characteristic words and text statements that are most associated with each cluster. For this report, five researchers (i.e., CV, IO, VM, SL, DWS) with expert domain knowledge interpreted the output of the algorithm separately to afterwards agree on the naming of the clusters.

Based on the output of Iramuteq [version 0.7, alpha 2] (20), a *χ*^2^ test of Independence was performed using R statistical software (19), to explore the association between the identified clusters and self-reported satisfaction ratings, i.e., positive vs. negative. A positive or negative hearing experience could be specified by the listener as part of the self-initiated EMAs, in the first screen of the survey.

## 3. Results

### 3.1. Demographics

**Figure 2:**
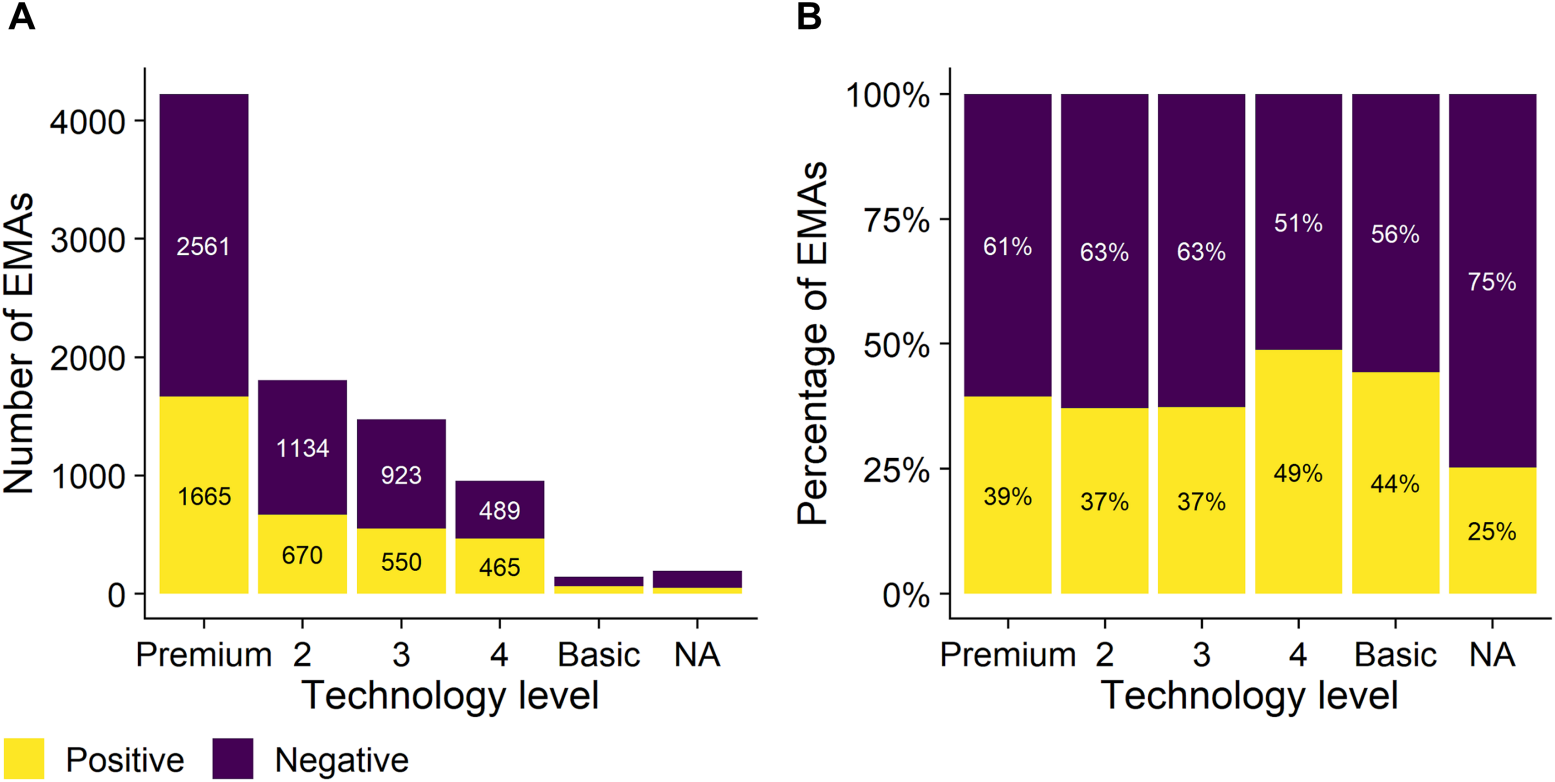
Number of self-initiated EMAs (*y*-axis; panel **A**) and proportion of self-initiated EMAs (*y*-axis; panel **B** – one column adds up to 100%) logged as a function of the technology level of the hearing devices that the smartphone app was connected to (*x*-axis). Five different technology levels were considered, ranging from premium (left) to basic hearing aids (right) in descending order. Yellow and purple bars represent positive and negative self-reported satisfaction ratings, respectively.

The 8,793 self-initiated EMAs were logged through a mobile application by 2,301 individual hearing aid wearers. The data was skewed: the sample contained between 1 and 4 EMAs for 78% of listeners (median (IQR) = 2 (1–4) EMAs per listener) and between 4 and 90 EMAs for 22% of listeners (min = 1; max = 90 EMAs per listener). Most EMAs included in the sample were logged for hearing aids fitted in the United States of America (5,577; 63%) and Canada (1,349; 15%), followed by Australia (945; 11%), England (893; 10%), New Zealand (19; 0.2%), and Ireland (10; 0.1%). Almost half of the EMAs (4,226; 48%) were logged for hearing aids with premium technology levels (see Figure 2). Overall, listeners indicated 3,462 positive experiences with hearing technology (39%) and 5,331 negative experiences (61%).

### 3.2. Cluster analysis

Across the 8,793 text statements considered in this analysis, with one text segment collected as part of one listener self-initiated EMA, a total number of 145,926 word forms and 6,245 different word forms were recorded. About 44% of all individual word forms (2,769 out of 6,245), corresponding to 2% of the total number of words (2,769 out of 145,926) were recorded only once.

**Figure 3:**
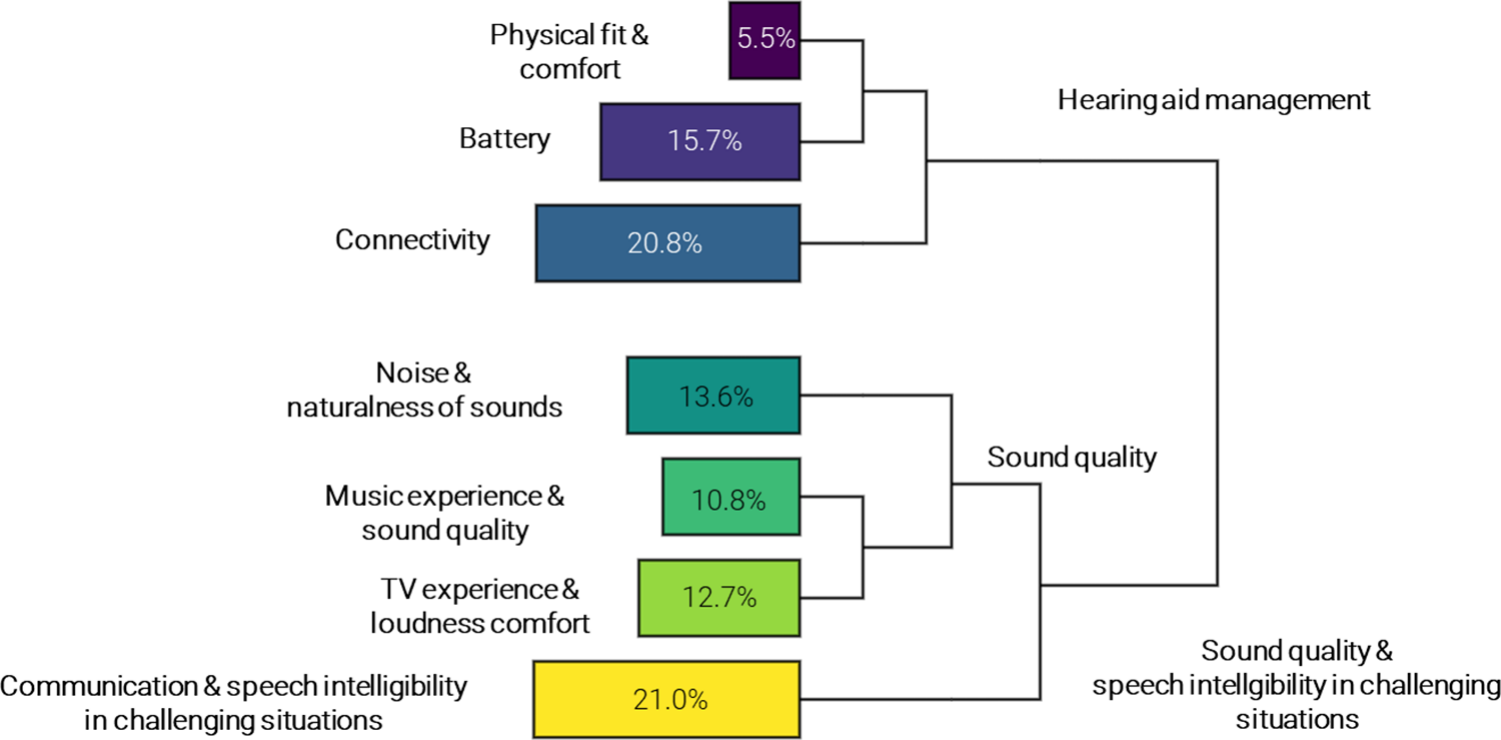
Graphical overview of the outcomes of the cluster analysis. The lines visualize the structure of the clustering, i.e., the two main branches on the right (“hearing aid management” and “sound quality & speech intelligibility in challenging situations”) and subsequent bipartitions, resulting in seven clusters. The percentages in the bars represent the size of each cluster. The clusters were named by the researchers based on characteristic words and text segments.

**Figure 4:**
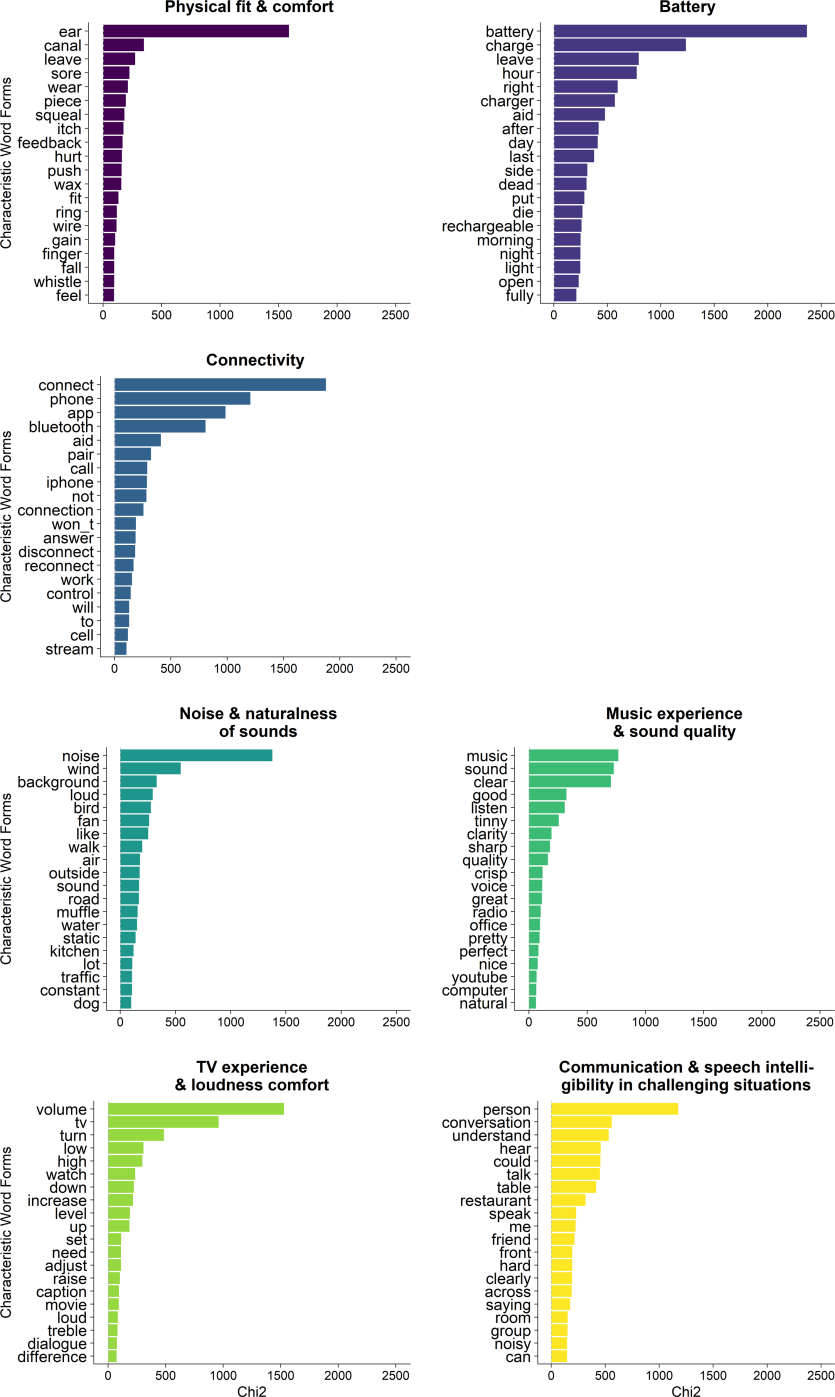
Graphical overview of the top twenty characteristic words for each cluster (*y*-axis), ranked from highest to lowest *χ*^2^-value (*x*-axis).

Cluster analysis revealed that 97.9% (8,606 out of 8,793) of the available text data could be classified into the resulting clusters, indicating that the results are representative of the corpus. The clustering revealed two branches (see Figure 3), one consisting of four clusters and the other consisting of three clusters. The branches and clusters were named by the researchers based on their expert domain knowledge, while interpreting the characteristic words (see Figure 4) and characteristic texts (see Table 1) derived for each cluster, based on a ranking of *χ*^2^-values.

**Table 1:** Top three characteristic texts examples for the resulting clusters, based on the highest *χ*^2^-values. Each example is an open-text statement provided by one hearing aid wearer during one EMA session.

Branch	Cluster (cluster size in %)	Characteristic text examples
Hearing aid management	Physical fit and comfort (5.5%)	– It is physically uncomfortable to wear my hearing aid I love being able to hear better but I had to stop wearing it yesterday because my ear was hurting so much even this morning I can’t put it back in because my ear is so sore it feels like my ear canal is all stretched or maybe swollen and so the ear piece feels too big it's quite uncomfortable I brought it up at my last appointment but it has gotten much worse – The left ear piece is uncomfortable my ear is sore and itchy also my ear feels blocked right is better also the behind the ear part is bulky and pushes my ears out away from my head – Left hearing aid keeps squealing I can push it in to the ear canal and it stops maybe the new ear bud is too loose
	Battery (15.7%)	– *P*ut left rechargeable battery into right hearing aid and charged overnight right battery then held the charge for only a few hours that battery has held a charge for fewer and fewer time day after day thousands confirming the nerf to replace the rechargeable batteries have resorted to utilizing regular batteries definitely need to replace rechargeable batteries for second time no idea why except for possibly the charging device is not functional but one of the rechargeable batteries seems to properly hold a charge regardless of which side is used in the charging device – Left side rechargeable battery just went dead the right side just gave me the low battery warning tone I have only had them on for 4 h after a full overnight charging I’m going to put them back into the charger – *P*ut in aids today at 0,930 working fine but now its 1,735 and left side battery done for day right still on going to put both on charge 1740 h
	Connectivity (20.8%)	– Devices are paired with new [phone brand] phone calls and media are streamed *via* Bluetooth but devices do not connect in the app to control volume selection of alternate modes like conversation in noise is also impossible is there a setting in the new phone or latest [software] that needs adjustment – The app isn’t Bluetooth connecting to my hearing aids my Bluetooth phone connection isn’t working when I’m making calls – Left hearing aid will not connect to the phone app for volume adjustment I disabled the Bluetooth phone connection but that didn’t work either
Sound quality and speech intelligibility in challenging situations	Noise and naturalness of sounds (13.6%)	– Having stepped outside to walk between buildings the left hearing aid made a noise that sounded like wind noise then all background noise become highly exaggerated and fairly extreme once I got inside the building I attempted to adjust the volume with the phone app the left hearing aid was unresponsive all sounds were highly exaggerated and I rebooted it sound returned to normal – Tried the car with speech program on the 1 h drive to and from [city] and the stables in [city] a lot of background noise but I seemed to detect the higher sensitivity in the right ear this background noise kind of like wind noise would alternately come and go not enough of a pattern to time the cycles or not enough of a distraction – Even in [shop] all I hear is a static I guess ac running sounds like the wind noise driving in car
	Music experience and sound quality (10.8%)	– Background music in action scene drowns voices background music also sounds bass heavy and all over the room voice sounds far away and not clear – Music seems to be crisper clearer enjoying working with the various sound levels and the equalizer – My music sounded great very clear
	TV experience and loudness comfort (12.7%)	– I rented a movie on demand and I could understand the dialogue pretty well it seems I am watching all my tv now with the aids volume turned up to 5 high then depending on the program I adjust the volume on the tv my husband says the tv is at a normal volume sometimes I will still miss a line and I will have to turn up the tv temporarily to hear a particular part then I adjust the tv volume down again some programs seem loud to me with my aid on 5 but I need them that way to understand the words – I can hear the tv better but have to make sure the volume in both ears are turned to the highest level it's the same for all the different environments – I turned my hearing aids up but dialogue was still hard to understand it was an [country] movie so accents were not a problem for me the tv volume was up to level 50 and after the movie I checked hearing aid volume it was at level 4 no wonder the clock ticking was too loud and its chiming was very uncomfortable
	Communication and speech intelligibility in challenging situations (21.0%)	– Used the places of worship setting when in the office last week the change to background noise made during my last visit really reduced the hissing which was great my only complaint is that I have to have the volume up at maximum to allow me to be able to understand people when they are talking through masks however at that volume I pick up on so much noise and sometimes if there are multiple conversations going on around me i can find it hard to hear the conversation I am having as it is drowned out by the other nearby conversations not sure if this can be easily rectified – Clear speech from all areas of large boardroom even from speakers not directly in front of me sitting a few rows ahead with back towards me easier to understand presentation also able to understand person in a side conversation after meeting amongst moderate noise from other groups talking – I was in a noisy bar restaurant seated at a rectangular table with 7 people I couldn’t hear people much of the time I could understand people directly across from me when I watched their lips and leaned in to hear

The largest of the two branches, containing 58.1% of clustered texts, was named *sound quality and speech intelligibility in challenging situations*. This branch consisted of four clusters, reported here from largest to smallest:

1. *Communication and speech intelligibility in challenging situations*, containing 21.0% of clustered texts, describing experiences related to conversations and speech understanding in social or acoustically challenging settings, such as restaurants, bars, office spaces, and cars – very often portraying multiple speakers in different locations.
2. *Noise and naturalness of sounds*, containing 13.6% of clustered texts, describing experiences related to the sound quality, and especially naturalness of sounds and different types of (background) noise, such as sounds of wind, air condition, or running water.
3. *TV experience and loudness comfort*, containing 12.7% of clustered texts, describing experiences related to watching TV and especially, volume settings when watching TV.
4. *Music experience and sound quality*, containing 10.8% of clustered texts, describing experiences related to listening to or streaming music, and especially, the sound quality of music – often related to clarity, timbre, and bass or treble volume.

The last three clusters were more related to each other than to the *communication and speech intelligibility in challenging situations* cluster, as revealed by the hierarchy of the bipartitions in the clustering (see Figure 3). The three clusters together were named *sound quality* clusters.

The smallest of the two branches, containing 42% of clustered texts, was named *hearing aid management*. This branch consisted of three clusters, reported from largest to smallest:

1. *Connectivity*, containing 20.8% of clustered texts, describing experiences related to different types of connectivity, e.g., pairing hearing aids with phones and mobile applications, or with other devices, such as TVs or tablets.
2. *Battery*, containing 15.7% of clustered texts, describing experiences related to rechargeable or disposable batteries for hearing aids, rechargeable hearing aids, and battery consumption of hearing aids.
3. *Physical fit and comfort*, containing 5.5% of clustered texts, describing experiences related to how the earpiece or hearing aid fits in the ear canal or around the pinna, as well as experiences related to feedback or whistling of the hearing aid, and cerumen build-up.

### 3.3. Association between identified clusters and self-reported satisfaction ratings (positive vs. negative)

**Figure 5:**
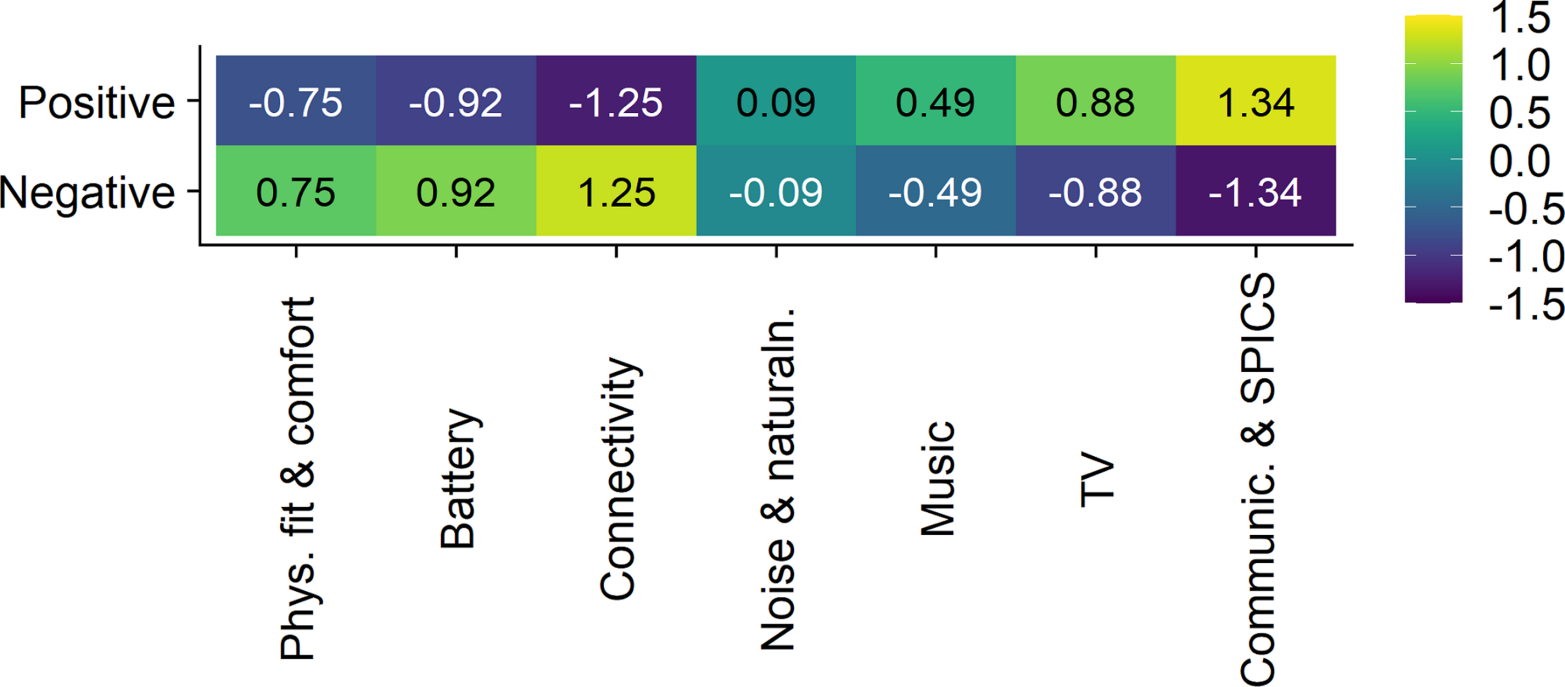
Heatmap visualizing the relationship between the seven clusters, i.e., the output of the cluster analysis (*x*-axis), and the ratio of positive vs. negative self-reported satisfaction ratings (*y*-axis). The values in the cells represent the standardized residuals of the two-sided *χ*^2^ Test of Independence. Their signs inform about the direction of the outcomes, with positive values (represented by green to yellow background colors) indicating a positive relationship and negative values (represented by blue to purple background colors) indicating a negative relationship. The names of the seven clusters on the *x*-axis are abbreviated. Their full names are, from left to right, “physical fit and comfort”, “battery”, “connectivity”, “noise and naturalness of sounds”, “music experience and sound quality”, “TV experience and loudness comfort”, “communication and speech intelligibility in challenging situations”.

A two-sided *χ*^2^ Test of Independence revealed that there was no significant relationship between the seven clusters and the ratio of positive vs. negative self-reported satisfaction ratings (*X^2^* (6) = 4.79, *p* = .57). The signs of the *χ*^2^ test's standardized residuals inform about the direction of the outcomes, with positive values suggesting a positive relationship, and negative values a negative relationship. As visualized in Figure 5 (first three columns, bottom row), the positive residuals suggested a trend for text statements related to hearing aid management to be associated with negative satisfaction ratings (z_physical fit & comfort _= .75, z_battery_ = .92, z_connectivity_ = 1.25). In turn, and as visualized in Figure 5 (last four columns, top row), text statements related to sound quality and speech intelligibility in challenging situations tended to be associated with positive satisfaction ratings (z_noise & naturalness of sounds_ = .09, z_music experience and sound quality _= .49, z_TV experience and loudness comfort_ = .88, z_communication & speech intelligibility in challenging situations _= 1.34). Note that a positive residual, suggesting a positive relationship between a cluster and positive satisfaction ratings, is accompanied by a negative residual of the same size, suggesting a negative relationship between the same cluster and negative satisfaction ratings.

## 4. Discussion

The aim of this retrospective analysis was to explore the use of written open-text statements, collected through self-initiated, smartphone-based EMAs as part of adult hearing care. In particular, we aimed to identify emerging themes in 8,793 reports of hearing aid wearers, describing daily life, in-the-moment experiences with hearing technology in their own words. We also explored the relationship between the identified themes and self-reported satisfaction ratings (positive vs. negative). Results revealed that almost 60% of listeners’ reports related to sound quality and speech intelligibility in challenging situations. In comparison, close to 40% of reports related to hearing aid management (as visualized by the two main branches in Figure 3). Sound quality and speech intelligibility-related reports tended to be associated with positive experiences, while reports describing hearing aid management tended to be associated with negative experiences (Figure 5).

The individual clusters identified (also see Figure 3) align with a subgroup of factors identified in a recent systematic review of qualitative studies (38), synopsizing hearing aid experiences in adults during and post hearing aid fitting. The factors identified by Oosthuizen et al. (38), such as difficulties experienced in background noise and group conversations, handling and continuous care, difficulties relating to physical fit, as well as sound quality issues, were reported as the most prevalent hearing device-related factors leading to sub-optimal hearing aid use. As indicated by the authors, these factors contrast consumer surveys showing high satisfaction with hearing aids and their features (39). Interestingly, close to 40% of the listeners' reports presented here were valued by listeners as positive experiences as well, even though we had hypothesized listeners to rely on the EMA mobile application primarily to request help from a clinician, reporting problems, or in-the-moment negative experiences.

As is apparent from Figure 2, the ratio of positive vs. negative experiences was similar for the three highest technology levels of devices (approximately 40% positive vs. 60% negative self-reported satisfaction ratings), but slightly more balanced (closer to 50% positive vs. 50% negative ratings) for the two more basic hearing device categories. As EMAs were mainly collected for higher technology levels (Figure 2, panel A), sample sizes were small for the latter two categories. There was no significant relationship between the ratio of positive vs. negative experiences and the identified clusters. Yet, all three hearing aid management clusters were more associated with negative experiences, while all four sound quality and speech intelligibility in challenging situations-related clusters were more associated with positive experiences (see Figure 5). The following anecdotal examples illustrate positive experiences related to sound quality and communication in difficult settings and highlight the detail with which these are described, e.g., “*can hear perfectly. husband is happy for me and him!! I can hear the wood floor in the hall and guest bath creak when I walk through and other small noises I had forgotten about.”, “TV turned down wife happy”, “I can hear certain tones in music now. it sounds so much sweeter.”, “love hearing my granddaughters voice”, “I attended an important work meeting with 8 client representatives and I could relax as hearing was so much easier and made the meeting much less stressful.”* As communication and speech intelligibility in challenging situations are often reported as persisting challenges for hearing aid wearers (38, 39), the positive experiences reported through self-initiated EMAs show benefits of hearing technology in these situations as well. Not unexpectedly, the reports often describe social activities or situations where significant others or frequent communication partners are present, e.g., “*My husband and I walked in the neighborhood and the wind was 13 miles per hour, which was strong. I could still hear my husband fine.”*, “*I could hear my granddaughter on the phone, it melted my heart!!”, “I walked with a colleague down a busy street and had no problem at all talking with her. That experience normally requires every ounce of my energy, and involved my saying “what?” a lot.”* In addition to improved hearing and communication (1, 38), psychosocial improvements are indeed among the most prevalent benefits of hearing aid use reported (38), e.g., increased interaction and participation in social situations (40–42), and greater confidence during communication (42, 43). Interestingly, we also encountered some statements entered by significant others describing experiences of a loved one with hearing aids, e.g., “*[name] was very comfortable in church with the conversation level, he also had a meeting and was able to hear and take notes during the meeting”.* This exemplifies how the use of EMA could foster family-centered care (18). It is indeed well-known that support from significant others (e.g., family members) can facilitate help-seeking for hearing loss (44, 45), adopting (46), successfully using, and being satisfied with hearing aids (47–49).

In addition to device-related factors, Oosthuizen et al. (38) identified factors related to the hearing aid user and hearing care professional in their systematic review, which can either positively or negatively impact hearing aid use and experiences. For example, the patient”s attitude towards hearing aids, expectation management, practical handling skills training, and patient-centered information counselling. We did not identify person-related themes in the cluster analysis presented here. While it is unclear how clinicians introduced hearing aid wearers to the EMA-functionality used for the data collection, it is possible that listeners were asked or were more inclined to describe in-the-moment device- rather than person-related experiences. Also, the clinical data was collected through a feature of a mobile application that was activated in the fitting software by a clinician, thereby likely not capturing experiences prior to hearing aid fitting. Even so, individual text statements from the current dataset provide anecdotal evidence for the person-related themes identified by Oosthuizen et al. (38). Some reports, for example, describe experiences regarding information counselling and practical handling skills, e.g., “*I”m wondering how long the aids last with rechargeable batteries. Can they be replaced if they no longer charge? (…)”, “I unpaired my hearing aids and now I don't know how to pair them again help!!!”, “it would be nice to listen to music off my phone at times like a pair of ear buds. if these do it please let me know how to do it.”,* Other reports describe experiences regarding expectations, e.g., “*(…) Saw a singer in the pub at night. It was hard to make out what people were saying. Went back to lip reading. Maybe I was expecting more.”, “My first experience in noisy restaurant. They were disappointing. They need to “focus” more on the person speaking. I was about a meter away.”, “wind noise more than I expected when driving my golf cart”.* Since this cluster analysis relies on identifying lexical themes based on cooccurring words, other types of analyses might shed more light on person-related themes specifically, such as sentiment analysis or qualitative content analysis (e.g. (31)).

In terms of distribution of reports across the clusters, about one fifth of listeners' experiences presented here were associated with communication and speech intelligibility in challenging situations. Another 37% of reports described elements of sound quality, which were captured in three separate clusters, i.e., noise and naturalness of sounds (13.6% of reports), TV experience and loudness comfort (12.7% of reports), and music experience and sound quality (10.8%). It is indeed known that, beyond speech intelligibility, communication, and sound quality (38, 39), watching TV (50) and listening to music are important for persons with hearing loss (51, 52). While hearing aid wearers might experience difficulties when watching TV or listening to music (50–52), not all of them are reported in the clinic (51, 53). In fact, listeners generally tend to underreport device-related problems in the clinic (53). New approaches, such as collecting EMAs as part of clinical practice through smartphone applications as described here, can lower the threshold to reporting such experiences. Allowing hearing aid users to provide open-text statements, further, can be particularly valuable, as it fosters reports that are more independent of predefined jargon or how survey questions are formulated. It allows listener to describe what is truly on their mind, and as shown here, can reveal detailed reports of daily-life situations and interactions with communication partners, providing unique insights into integral aspects of hearing care. Still, we must be mindful of how new technologies can introduce new challenges. In addition to known hearing aid handling matters such as physical fit and comfort (5.5% of reports) or battery-related experiences (15.7% of reports), another one fifth of listeners' reports described experiences related to different types of connectivity and pairing of hearing aids with Bluetooth-connected devices (20.8% of reports). Additional troubleshooting and even frustration with smartphone-connected hearing aids due to connectivity and pairing have indeed previously been described (54).

### 4.1. Limitations

To our knowledge, this is the first study to report on a large data set of self-initiated EMAs, containing open-text statements provided by hearing aid wearers. It is important to acknowledge that the data explored was not collected for research purposes. The data was logged to support hearing aid wearers and clinicians during the hearing rehabilitation process, i.e., for purposes of counselling and fine-tuning by collecting in-the-moment feedback. Using clinical data for research purposes offers real-world insights that can support continuous improvements in health care (55) and personalized care (56), but comes with limitations. Verheij et al. (57) and Dillard et al. (58), for instance, describe the use of electronic health records for research purposes and how each step in the process, from a clinically relevant event (e.g., decision of an individual to seek help, or decision of a clinician to perform a diagnostic test) to data cleaning can impact the quality of such data sets and generalizability of the results. Thus, it is important to note that the feature through which the present data set was collected was available only to listeners who actively managed their hearing loss through hearing aids, who chose a particular brand of hearing aids, who were comfortable using a mobile application, for whom clinicians had activated the feature (e.g., when they thought the person could benefit from or would be motivated to use a real-time feedback system), and who described their experiences in English. Further, participants self-selected to open the app to provide an optional open-text statement. As a result, the data presented here is likely representative of a tech savvy and motivated subgroup of hearing aid wearers. When considering the initial sample of 30,127 self-initiated EMAs prior to data cleaning, it is indeed noteworthy that 60% (18,170 EMAs) included an optional open-text statement – which can be considered a high response rate for an optional assessment. Still, the majority of listeners (78%) only used the real-time feedback system between one and four times. While the results suggest that self-initiated EMAs can provide valuable audiological insights into the experiences of, at least a subgroup, of hearing aid wearers, EMA is prone to poor compliance when participants are asked to determine themselves when an event of interest takes place to, in turn, self-initiate a response (16). Therefore, it would be of interest for future research to investigate compliance to EMA in a large, clinical population of hearing aid wearers when, e.g., time- or event-based triggering is introduced.

A further limitation of this study is that we cannot rule out that listeners self-initiated EMAs retrospectively, once the hearing event was over, thereby still being prone to recall bias. As is apparent from anecdotal examples described in the past tense, listeners might especially describe events retrospectively when they occurred in a setting where phone use is less appropriate, e.g., “*saw a singer in the pub last night (…)”,* “*I attended an important work meeting with 8 client representatives (…)”.* At the same time, it is encouraging that listeners still provided feedback shortly after the event, e.g., when they had time or felt comfortable doing so, as the information could still be valuable to clinicians. Finally, no demographic or audiological information was available for the hearing aid wearers, beyond the hearing aid technology level.

## 5. Conclusions

The use of smartphone technology can provide an effective means to bring real-life and (near-)real-time feedback from hearing aid wearers into the clinic. This first report of open-text statements, collected through self-initiated smartphone-based EMAs, shows that listeners reported experiences related to communication in challenging situations, sound quality dimensions, and hearing aid management. While reports of communication in challenging situations and sound quality tended to be valued positively, experiences related to hearing aid management tended to be valued negatively. Anecdotal examples, further, revealed very detailed reports of daily-life situations, nuances, and interactions with significant others that provide unique insights into integral aspects of hearing care. Thereby, this study illustrates that, while EMA can come with a participant burden, at least a subsample of motivated real-world hearing aid wearers could use their own words to provide valuable feedback to a clinician through self-initiated EMA, to inform more responsive, personalized, and family-centered hearing care. Also, it suggests opportunities for large-scale data collections to facilitate training of machine learning algorithms fostering hearing technology to anticipate user needs.

## Data Availability

The datasets presented in this article are not readily available because of the mobile application's data privacy notice. Requests to access the datasets should be directed to Charlotte.Vercammen@sonova.com.
